# Effect of Different Induction Immunosuppression on the Incidence of Infectious Complications after Kidney Transplantation—Single Center Study

**DOI:** 10.3390/jcm13082162

**Published:** 2024-04-09

**Authors:** Matej Vnučák, Karol Graňák, Monika Beliančinová, Patrícia Kleinová, Tímea Blichová, Vladimír Doboš, Ivana Dedinská

**Affiliations:** 1 Transplant-Nephrology Centre, University Hospital Martin, Kollárova 2, 03601 Martin, Slovakia; vnucak.matej@gmail.com (M.V.); beliancinova.monika@gmail.com (M.B.); pata.kleinova@gmail.com (P.K.); timea.blichova@gmail.com (T.B.); ivana.dedinska@uniba.sk (I.D.); 2 1st Department of Internal Diseases, University Hospital Martin and Jessenius Medical Faculty of Comenius University, Kollárova 2, 03601 Martin, Slovakia; vladimirdobos@icloud.com

**Keywords:** kidney transplantation, thymoglobulin, basiliximab, infections, induction immunosuppression, thrombocytopenia

## Abstract

**Background/Objectives:** Potent immunosuppression lowers the incidence of acute graft rejection but increases the risk of infections. In order to decrease either infectious complications or acute rejection, it is necessary to identify risk groups of patients profiting from personalized induction immunosuppressive treatment. The aim of our analysis was to find whether there were higher incidences of infectious complications after kidney transplantation (KT) in groups with different induction immunosuppressive treatment and also to find independent risk factors for recurrent infections. **Materials:** We retrospectively evaluated all patients with induction treatment with basiliximab after kidney transplantation from 2014 to 2019 at our center relative to age- and sex-matched controls of patients with thymoglobulin induction immunosuppression. **Results**: Our study consisted of two groups: basiliximab (39) and thymoglobulin (39). In the thymoglobulin group we observed an increased incidence of recurrent infection in every observed interval; however, acute rejection was seen more often in the basiliximab group. A history of respiratory diseases and thrombocytopenia were identified as independent risk factors for recurrent bacterial infections from the first to sixth month after KT. Decreased eGFR from the first month, infections caused by multi-drug-resistant bacteria, and severe infections (reflected by the need for hospitalization) were identified as independent risk factors for recurrent bacterial infections from the first to the twelfth month after KT. **Conclusions**: We found that in the group of patients with thymoglobulin induction immunosuppressive treatment, infectious complications occurred significantly more often during the entire monitored period with decreased incidence of acute humoral and cellular rejection occurred more often.

## 1. Introduction

Induction immunosuppressive therapy is the gold standard in the treatment of kidney transplant recipients (KTRs) prior to kidney transplantation (KT) to prevent acute graft rejection. According to KDIGO guidelines, except for KT between identical twins, all KTRs need induction immunosuppression. The choice of induction immunosuppression is based on the immunological risk of the KTR. The first choice should be an interleukin 2 receptor antagonist (IL2-RA): basiliximab, a chimeric murine–human monoclonal antibody binding selectively to the high-affinity IL-2R [1,2]. Induction therapy achieves complete IL-2R suppression in 4–6 weeks in adult KTRs [3,4]. In KTRs with a higher risk of acute graft rejection (high immunological risk), induction with lymphocyte-depleting therapy is used. The most commonly used agent is rabbit antithymocyte globulin (rATG), which provides dose-dependent depletion of T cells [5]. Antilymphocyte therapies are associated with increasing rates of infections after solid organ transplantation (SOT) [6]. It is necessary to find appropriate strategies to prevent the risk of infection as the most frequent cause of graft loss after KT, on one hand, and the risk of biopsy-proven acute rejection (BPAR), on the other hand, by identifying risk factors increasing the risk of infection.

## 2. Materials and Methods

Our retrospective case control study included all kidney transplant recipients receiving basiliximab as an induction treatment at Transplant-Nephrology Center Martin, Slovakia, from 2014 to 2019. Basiliximab was given at a dosage of 20 mg intravenously on day 0 and 4 days after KT. Age- and sex-matched patients with induction treatment with rATG served as controls. rATG was given for 3 days, starting on day 0 (cumulative doses of 3.5 mg/kg). All patients received infection prophylaxis sulphamethoxazole/trimethoprim for 6 months and 3 months of CMV prophylaxis in the rATG group or in the basiliximab group if the donor was CMV positive and the recipient was CMV negative. A daily dose of 100 mg of fluconazole was given to all patients as mycotic infection prevention for 3 months. Prior to the surgical procedure, 1500 mg of cefuroxime was given intravenously as an initial dose and then 750 mg every 8 h, for a total of three doses.

The baseline information collected included the age of the recipients at the time of KT, sex, information about dialysis treatment (the type of dialysis and duration in months), the waiting list time in days, and the kidney donor type (living donor, standard donor criteria, and extended criteria donor defined as a donor older than 60 years or a donor older than 50 years with at least two of the following: cause of death from cerebrovascular accident, serum creatinine level > 133 µmol/L, and history of arterial hypertension). In our study, the levels of actual and maximal panel-reactive antibodies were monitored, as well as the cold ischemia time and the delayed graft function, defined as a need for dialysis seven days after KT. We also noted the presence of comorbidities: diabetes mellitus, ischemic heart disease, urinary tract infection (UTI) (history of recurrent UTI prior to KT), gastrointestinal disease (history of chronic gastritis and peptic ulcerations), respiratory diseases (chronic obstructive pulmonary disease and asthma bronchiale), autoimmune diseases, and a history of cancer. We monitored the duration of the urinary catheter placement and the duration of the ureteral stent placement in days.

We also monitored the incidence of acute graft rejection diagnosed by needle biopsy (BPAR) according to the 2019 Banff criteria and by de novo donor-specific antibody (dnDSA) detection using the Luminex method. We monitored the serum levels of creatinine, and we estimated the glomerular filtration rate (using the CKD-EPI creatinine equation in mL/s) at 3, 6, and 12 months after KT. We monitored dnDSA regularly or when graft function deteriorated. We performed a protocol kidney graft biopsy 3 months after KT.

We monitored the incidence of infectious complications twelve months after kidney transplantation. Infections were monitored within one month after KT (from day 0 to day 30), then until the 6th month and from the 6th to the 12th month after kidney transplantation. During the observed period, the levels of creatinine at the 1st, 6th, and 12th months after kidney transplantation (µmol/L) were recorded, and based on those, we estimated the glomerular filtration based on the CKD-EPI formula (mL/min/1.73 m^2^).

The incidence of infection according to the etiology (bacterial, fungal, viral, or CMV) was monitored. Infection was defined by either clinical or laboratory signs. A separate group of infections was CMV infection verified by PCR, when more than 1000 copies of virus/mL were considered to be a significant replication of CMV. We also monitored the incidence of infections by location and severity. The severity of the infection was reflected in the need for hospitalization, an intensive care unit, or vasopressoric support. Urogenital tract infection was diagnosed based on clinical signs and/or positive urine cultivation. Respiratory infection was diagnosed based on clinical signs and/or imaging methods (X-ray/CT-verified bronchopneumonia) and/or positive sputum or bronchoalveolar lavage cultivation. A blood infection was diagnosed based on clinical signs of infection and the presence of an infectious agent in the blood culture. Another infection included a local skin or wound infection. The sensitivity to individual antibiotics was determined in the case of a positive culture finding. Multidrug resistance was defined as the resistance to at least one antibiotic drug from at least three different antibiotic groups. In our study, we monitored the incidence of acute rejection verified histologically by needle graft biopsy or by the presence of de novo donor-specific antibodies (dnDSAs) detected by Luminex. The incidence of leukopenia, defined as a decrease in white blood cells below 3.9 × 10^9^/L, and the incidence of thrombocytopenia, defined as a decrease in platelets below 140 × 10^9^/L, were monitored with the lowest values of white blood cells and platelets recorded. We also monitored the need for granulocyte colony stimulating factors (G-CSF) given at 480 µg daily for three days when the absolute neutrophil count dropped below 0.5 × 10^9^/L.

A certified statistical program, MedCalc Software version 13.1.2 (MedCalc Software VAT registration number BE 0809 344,640, Member of International Association of Statistical Computing, Ostend, Belgium) was used for statistical analysis. Categorical variables were presented as weighted percentages and counts. Parametric (*t*-test) or non-parametric (Mann–Whitney) tests were used to compare continuous variables between groups. Using the χ^2^ test and Fisher’s exact test, we analyzed the associations between categorical variables. We used logistic regression for multivariate analysis for independent predictors of the incidence of infections. A statistically significant *p* value was considered to be <0.05.

Ethical approval: According to the ethical standards of the institutional ethical committee (University Hospital Martin), all the procedures involving human participants were approved. All signed informed consents were archived for at least 20 years after research was completed and was approved by the university hospital’s ethical committee. The clinical and research activities being reported were consistent with the principles of the Declaration of Istanbul, as outlined in the Declaration of Istanbul on organ trafficking and transplant tourism.

## 3. Results

During the study period, 78 kidney transplant recipients were included, including 39 patients with basiliximab and 39 patients with thymoglobulin induction immunosuppressive treatment. The basic characteristics of the patients, immunosuppression level, graft function, and laboratory parameters are shown in Table 1 and Table 2.

In the compared groups, there were statistically significant differences in serum creatinine level (*p* = 0.0241), eGFR (*p* = 0.0210), white blood cells (*p* = 0.009), the incidence of leukopenia (*p* = 0.024), and thrombocytopenia (*p* = 0.006) at the first month after KT (Table 3).

From the first to the sixth month after KT, in the thymoglobulin group, we observed a significant difference in the incidence of mycotic infection (*p* = 0.0409), UTI (*p* = 0.0384), sepsis (*p* = 0.0497), and leukopenia (*p* = 0.0384) compared with the basiliximab group (Table 4).

From the sixth to the twelfth month after KT, there were significant differences in the incidence of other infections (*p* = 0.0218) and severe infections reflected by the need for hospitalization (*p* = 0.0269) in the thymoglobulin group. However, in the basiliximab group, we observed an increased incidence of acute cellular (*p* = 0.0218) and acute humoral rejection (*p* = 0.0218) (Table 5).

We observed an increased incidence of recurrent infection in general (*p* = 0.0033), bacterial infections (*p* = 0.0008), infections caused by multi-drug-resistant bacteria (*p* = 0.0140), and a UTI (*p* = 0.0072) in the thymoglobulin group one month after KT. From the first to the sixth month after KT, there was an increased incidence of bacterial infections (*p* = 0.0416) caused by multi-drug-resistant bacteria (*p* = 0.0261) and a UTI (*p* = 0.0332) in the thymoglobulin group and a higher incidence of recurrent infections in general (*p* = 0.0224) and bacterial infections (*p* = 0.0466) in the thymoglobulin group from the sixth to the twelfth month after KT (Table 6).

In our study, we identified several risk factors for recurrent bacterial infections from the first to the sixth month after KT: a history of respiratory disease such as COPD or asthma bronchiale (OR 5.7200; *p* = 0.0062), the serum level of creatinine (OR 1.0074; *p* = 0.0054), and eGFR (OR 0.9739; *p* = 0.0048). Risk factors for the first month after KT and the sixth month after KT were the serum level of creatinine (OR 1.0111; *p* = 0.0012) and eGFR (OR 0.9672; *p* = 0.0069). Thrombocytopenia observed in the first to the sixth month after KT (OR 3.500; *p* = 0.0076), infections caused by multi-resistant bacteria (OR 7.4727; *p* < 0.0001), and the need for hospitalization (OR 10.000; *p* = 0.0007) (Table 7) were also risk factors.

By using logistic regression, we identified independent risk factors for the incidence of recurrent bacterial infections from the first to the sixth month after KT: a history of respiratory diseases (OR 3.8640; *p* = 0.0108), thrombocytopenia recorded in the first to the sixth month after KT (OR 9.5904; *p* = 0.0104), and infections caused by multi-resistant bacteria (OR 9.8942; *p* = 0.0003) (Table 8).

Risk factors for recurrent bacterial infections from the sixth to the twelfth month after KT were as follows: daily dose of MPA from the first to the sixth month after KT (OR 1.9987; *p* = 0.0319) and from the sixth to the twelfth month after KT (OR 1.9984; *p* = 0.0347); serum creatinine levels in the first to the sixth month after KT (OR 1.0116; *p* = 0.0004) and from the sixth to the twelfth month after KT (OR 1.0030; *p* = 0.0012); eGFR in the same intervals, from the first to the sixth month (OR 0.9620; *p* = 0.0044) and from the sixth to the twelfth month after KT (OR 0.9550; *p* = 0.0001); infections caused by multi-resistant bacteria (OR 5.000; *p* < 0.0001); and severe infections reflected by need for hospitalization (OR 2.2445; *p* < 0.0001) (Table 9). By using logistic regression, we identified infections caused by multi-drug-resistant bacteria as an independent risk factor for the incidence of recurrent bacterial infections from the sixth to the twelfth month after KT (OR 8.4263; *p* = 0.0055) (Table 10).

## 4. Discussion

Induction immunosuppression is an inseparable part of kidney transplantation in order to prevent acute graft rejection. It is necessary to evaluate immunological risk prior to kidney transplantation to choose adequate induction immunosuppression to prevent acute rejection on one hand and minimize the risk of side effects such as infections and malignancy on the other. In most prospective trials, rATG was not associated with an increased risk of bacterial infection compared with no induction or other induction therapies (IL-2RA) [7,8,9,10,11].

In our study, we found significant differences in the incidence of UTI, mycotic infection, and sepsis in the rATG group from the first to the sixth month after KT and an increased incidence of other infections (mainly skin and wound infections) and more severe infections requiring hospitalization. We also found statistically significant differences in the incidence of recurrent infections in the general first month and from the sixth to the twelfth month after KT in the rATG group, recurrent bacterial infection in every interval, an increased incidence of recurrent UTI 6 months after KT, and recurrent infections caused by MDR bacteria in the same period. rATG provides dose-dependent depletion of T cells; the risk of infection depends on the dosing strategy [5]. In our study, there was no significant difference in the daily dose of MPA or levels of TAC between the observed periods. It is generally known that the increasing incidence of infections after SOT has contributed to antilymphocyte induction therapies and intensified maintenance immunosuppression. A study by Branner et al. (2006) brought similar conclusions to those of our study. In the group of patients with ATG, the incidence of infections, UTIs, and bacterial infections was statistically significantly higher. BPAR was more frequent in the group of patients with basiliximab induction. However, the mentioned study does not identify risk factors for the emergence of infections or a critical interval for the emergence of a rejection episode. In our study, the incidence of ACR and AHR was statistically more frequent in the period from the sixth to the twelfth month after Kx [6]. In a randomized study by Martinez-Mier et al. (2021), there were no statistically significant differences in the incidence of infections between the groups of patients with ATG and basiliximab, despite the fact that the concentration of TAC was statistically significantly higher in the group of patients with ATG, which represents a risk factor for infections [12,13]. Pham et al. (2020) in their study identified an increased incidence of infections, bacterial infections, and UTIs in patients older than 65 years with induction treatment with ATG compared with basiliximab, where the only independent risk factor for the development of infections was induction treatment with ATG [14]. In our study, the mean age in both groups was 44.9 ± 11.9 years in the basiliximab group and 45.3 ± 11.6 years in the ATG group.

However, in the rATG group, we observed a significantly higher incidence of leukopenia. According to the study of Hanningsen et al. (2021), leukopenia was associated with an increased mortality rate, an increased incidence of bacterial and viral infections, more rejections, and the administration of rATG [15]. On the other hand, there was an increased incidence of acute cellular and humoral rejection in the basiliximab group from the sixth to the twelfth month after KT. In the past, the incidence of acute rejection was 10–30%; today, it is a maximum of 10% because of the implementation of protocol graft biopsy 3 months after kidney transplantation, when we can diagnose subclinical graft rejection [16].

By univariate analysis, we identified risk factors for recurrent bacterial infections from the first to the sixth month after KT: low eGFR in the first month after KT and from the first to the sixth month after KT, the incidence of MDR bacteria, the need for hospitalization, and thrombocytopenia diagnosed in the period of the first to the sixth month after KT.

A study by Su et al. (2018) found patients with lower eGFR have a higher risk of infections caused by MDR bacteria [17]. In our study, we identified low eGFR as a risk factor for recurrent bacterial infections twelve months after kidney transplantation. Therefore, in patients with low eGFR and other risk factors, we should focus on reducing the risk of developing infections and optimizing immunosuppressive therapy by intensively monitoring the level of immunosuppressive drugs and their effective doses, together with increased surveillance and more frequent checks in terms of the early diagnosis of bacterial infections, especially UTIs, and their subsequent treatment, together with other preventive measures. In correlation with other results of our study, a risk factor for infections from the first to the twelfth month after KT was also the occurrence of infections caused by MDR bacteria (verified by univariate and multivariate analysis); therefore, it is necessary to follow the basic principles of rational antibiotic treatment in order to avoid prophylactic ATB treatment during mini-invasive procedures or with asymptomatic bacteriuria in the early post-transplantation period [18].

Thrombocytopenia was found to be a risk factor for recurrent bacterial infections from the first to the sixth month after kidney transplantation. In the study by Qu et al. (2018), the authors found a low platelet count was correlated with infections in patients with primary immune thrombocytopenia and was also an independent risk factor for the infections [19]. Recent research has shown that platelets can modulate innate and adaptive immune responses, which leads to protein production such as fibrinogen, C-reactive protein, and complement proteins. Platelets are also known as a major source of interleukin-1β produced upon platelet stimulation and inducing the acute phase response to infection [20]. Platelets can inhibit bacterial growth, affect white blood cell recruitment and functions, affect cytokine responses, and activate the coagulation system and vascular endothelium [21,22,23,24,25]. Thrombocytopenia is a surrogate marker for poor prognosis, especially for patients with sepsis [26]. This was also confirmed in our study, when a low platelet count was an independent risk factor for recurrent bacterial infections from the first to the sixth month after KT.

The daily dose of MPA was also identified as a risk factor for recurrent bacterial infection from the first to the twelfth month after KT. This was confirmed in another study of our center, when a daily dose of MPA >1080 mg was identified as an independent risk factor for recurrent infection starting in the first month after KT, with a significant association between the incidence of infections and the daily dose of MPA without an increased risk of acute graft rejection [11].

In our study, we identified the history of respiratory diseases (asthma bronchiale and chronic obstructive pulmonary disease) as an independent risk factor for recurrent bacterial infections from the first to the sixth month after KT. A study by Bhat et al. (2015) investigated the phenotype of immune cells in patients with COPD and found extensive immune dysfunction due to the presence and functional activity of T regulatory cells [27]. In the first six months, the patient is at the greatest risk of developing infections, and it is the history of respiratory diseases in the context of immune dysregulation that can play a contributing factor in conjunction with other risk factors (as proven in our study) to an increased susceptibility to recurring infections.

In our study, we found that in the group of patients with thymoglobulin induction immunosuppressive treatment, infectious complications occurred significantly more often during the entire monitored period. On the other hand, in the group of patients receiving induction treatment with basiliximab, acute humoral and cellular rejection occurred more often. The subject of further research should be personalized induction, as well as maintenance immunosuppressive treatment, with the identification of patients at higher infection risk who would benefit from induction treatment with basiliximab (without the simultaneous increased risk of rejection episodes) and, at the same time, the identification of patients with an increased risk of rejection who would benefit from a more potent induction immunosuppressive treatment (thymoglobulin) without an increased risk of infectious complications. We can consider impaired graft function (reflected by lower eGFR), a daily dose of MPA > 1080 mg, thrombocytopenia, and infections caused by MDR bacteria as factors leading to an increased risk of recurrent infections.

## Figures and Tables

**Table 1:** The basic characteristics of the patients.

	Basiliximab n = 39	Thymoglobulin n = 39	*p* Value
**Males (%)**	71.8	71.8	1.0000
**Age (years)**	44.9 ± 11.9	45.3 ± 11.6	0.8809
**Peritoneal dialysis (%)**	5.1	5.1	1.0000
**Dialysis time (months)**	21 ± 19	28 ± 28	0.2003
**Duration on WL (days)**	242 ± 248 (median 165)	290 ± 453 (median 128)	0.5633
**ECD (%)**	25.6	5.1	**0.0126**
**Living donor (%)**	5.1	0	0.1558
**PRA actual (%)**	1 ± 1 (median 0)	1 ± 3 (median 0)	1.0000
**PRA maximal (%)**	2 ± 5 (median 0)	4 ± 11 (median 1)	0.3046
**CIT (min)**	561 ± 295	661 ± 239	0.1041
**DGF (%)**	5.1	12.8	0.2366
**BMI (kg/m^2^)**	27 ± 6	26 ± 5	0.4264
**Diabetes mellitus (%)**	17.9	20.5	0.7721
**Urinary tract disease (%)**	30.8	7.7	**0.0102**
**Ischemic heart disease (%)**	12.8	23.1	0.2390
**Gastrointestinal disease (%)**	10.3	33.3	0.0145
**Respiratory disease (%)**	5.1	30.8	**0.0033**
**Autoimmune diseases (%)**	7.7	5.1	0.6412
**Cancer in anamnesis (%)**	5.1	7.7	0.6412

BMI—body mass index; CIT—cold ischemia time; DGF—delayed graft function; ECD—extended criteria donor, PRA—panel reactive antibody, WL—waiting list.

**Table 2:** Comparison of basiliximab and thymoglobulin group in general.

	Basiliximab n = 39	Thymoglobulin n = 39	*p* Value
**Cyclosporine A treatment (%)**	2.6	2.6	1.0000
**Corticosteroid treatment (%)**	100	100	1.0000
**ACR (%)**	17.9	17.9	1.0000
**AHR (%)**	23.1	15.4	0.3915
**Leukopenia (%)**	56.4	79.5	**0.0299**
**Thrombocytopenia (%)**	66.7	66.7	1.0000
**CSF (%)**	2.6	20.5	0.0140
**Urinary catheter (days)**	6 ± 3	5 ± 3	0.1453
**JJ urethral stent (days)**	47 ± 10	49 ± 21	0.5928
**Positive baceterial cultures (early postoperative period)**			
**Urinary catheter (%)**	56.4	74.4	0.0969
**Urine (%)**	15.4	15.4	1.0000
**Central venous line (%)**	30.8	33.3	0.8142
**Abdominal drainage (%)**	43.6	53.8	0.3706
**Other (%)**	12.8	2.6	0.0932
**Blood culture (%)**	2.6	2.6	1.0000

ACR—acute cellular rejection; AHR—acute humoral rejection; CSF—colony stimulating factor.

**Table 3:** Comparison of basiliximab and thymoglobulin group at the first month after kidney transplantation.

	Basiliximab n = 39	Thymoglobulin n = 39	*p* Value
Mean TAC level 1M (ng/mL)	14.6 ± 4.3	13.4 ± 3.0	0.1570
MPA daily dose M1 (mg/day)	1145 ± 308	1218 ± 401	0.3701
**Creatinine level M1 (µmol/L)**	153 ± 47	212 ± 153	**0.0241**
**eGFR M1 (mL/min)**	50.3 ± 16.8	40.9 ± 18.4	**0.0210**
**WBC M1 (10^9^/L)**	5.3 ± 2.2	3.8 ± 1.6	**0.0009**
Platelets M1 (10^9^/L)	137 ± 46	130 ± 57	0.5524
Infection in general (%)	38.5	53.8	0.1781
Bacterial infection (%)	35.9	48.7	0.2556
Viral infection (%)	7.7	2.6	0.3113
Mycotic infection (%)	0	2.6	0.3139
Parasitic infection (%)	0	5.1	0.1558
CMV viremia (%)	0	0	1.0000
Multi-drug-resistant bacteria (%)	20.5	38.5	0.0833
UTI (%)	30.8	43.6	0.2453
RTI (%)	5.1	5.1	1.0000
GI infection (%)	5.1	0	0.1558
Sepsis (%)	0	2.6	0.3139
Other (%)	2.6	12.8	0.0932
Need for hospitalization (%)	0	5.1	0.1558
ICU (%)	0	0	1.0000
Septic shock (%)	0	0	1.0000
ACR (%)	7.7	10.3	0.6902
AHR (%)	15.4	12.8	0.7431
**Leukopenia (%)**	28.2	53.8	**0.0224**
**Thrombocytopenia (%)**	61.5	23.1	**0.0006**
CSF (%)	0	2.6	0.3139

WBC—white blood cells; TAC—tacrolimus; M—month; MPA—mycophenolic acid; eGFR—estimated glomerular filtration rate; CMV—cytomegalovirus; UTI—urinary tract infection; RTI—respiratory tract infection; GI—gastrointestinal; ICU—intensive care unit; ACR—acute cellular rejection; AHR—acute humoral rejection; CSF—colony stimulating factor.

**Table 4:** Comparison of basiliximab and ATG groups from first to sixth month after kidney transplantation.

	Basiliximab n = 39	Thymoglobulin n = 39	*p* Value
Mean TAC level M6 (ng/mL)	9.5 ± 1.5	9.6 ± 1.8	0.7906
MPA dose M6 (mg/day)	646 ± 362	655 ± 481	0.9259
Creatinine level M6 (µmol/L)	153 ± 62	175 ± 102	0.2533
eGFR M6 (mL/min)	52.6 ± 19.5	47.5 ± 19.9	0.2566
WBC M6 (10^9^/L)	3.9 ± 1.6	3.2 ± 1.7	0.0650
Platelets M1 (10^9^/L)	157 ± 49	160 ± 57	0.8038
Infection in general (%)	89.7	89.7	1.0000
Bacterial infection (%)	56.4	76.9	0.0564
Viral infection (%)	38.5	28.2	0.3378
**Mycotic infection (%)**	0	10.3	**0.0409**
Parasitic infection (%)	0	0	1.0000
CMV viremia (%)	48.7	28.2	0.0645
Multi-drug-resistant bacteria (%)	48.7	59	0.3647
**UTI (%)**	48.7	71.8	**0.0384**
RTI (%)	15.4	30.8	0.1089
GI infection (%)	12.8	10.3	0.7315
**Sepsis (%)**	2.6	15.4	**0.0497**
Other (%)	15.4	25.6	0.2676
Need for hospitalization (%)	12.8	23.1	0.2390
ICU (%)	0	7.7	0.0791
Septic shock (%)	0	0	1.0000
ACR (%)	7.7	7.7	1.0000
AHR (%)	7.7	2.6	0.3113
**Leukopenia (%)**	48.7	71.8	**0.0384**
Thrombocytopenia (%)	43.6	41	0.8174
CSF (%)	2.6	17.9	0.2971

ACR—acute cellular rejection; AHR—acute humoral rejection; CMV—cytomegalovirus; CSF—colony stimulating factor; eGFR—estimated glomerular filtration rate; GI—gastrointestinal; ICU—intensive care unit; M—month; MPA—mycophenolic acid; RTI—respiratory tract infection; TAC—tacrolimus; UTI—urinary tract infection.

**Table 5:** Comparison of basiliximab and ATG group from sixth to twelfth month after kidney transplantation.

	Basiliximab n = 39	Thymoglobulin n = 39	*p* Value
Mean TAC level M12 (ng/mL)	6.5 ± 1	5.8 ± 2	0.0543
MPA daily dose M12 (mg/day)	628 ± 295	582 ± 410	0.5712
Creatinine level M12 (µmol/L)	139 ± 63	159 ± 90	0.2591
eGFR M12 (mL/min)	62.1 ± 21.9	54.2 ± 22.7	0.1219
WBC M12 (10^9^/L)	5.5 ± 1.8	5.3 ± 1.9	0.6346
Platelets M12 (10^9^/L)	170 ± 45	181 ± 54	0.3315
Infection in general (%)	69.2	74.4	0.6121
Bacterial infection (%)	43.6	56.4	0.2614
Viral infection (%)	23.1	23.1	1.0000
Mycotic infection (%)	2.6	7.7	0.3113
Parasitic infection (%)	0	0	1.0000
CMV viremia (%)	20.5	23.1	0.7823
Multi-drug-resistant bacteria (%)	20.5	38.5	0.0833
UTI (%)	35.9	53.8	0.1143
RTI (%)	20.5	25.6	0.5952
GI infection (%)	10.3	10.3	1.0000
Sepsis (%)	0	7.7	0.0791
**Other (%)**	0	12.8	**0.0218**
**Need for hospitalization (%)**	2.6	17.9	**0.0269**
ICU (%)	0	5.1	0.1558
Septic shock (%)	0	2.6	0.3139
**ACR (%)**	12.8	0	**0.0218**
**AHR (%)**	12.8	0	**0.0218**
Leukopenia (%)	15.4	20.5	0.5599
Thrombocytopenia (%)	23.1	17.9	0.5307
CSF (%)	0	0	1.0000

ACR—acute cellular rejection; AHR—acute humoral rejection; CMV—cytomegalovirus; CSF—colony stimulating factor; eGFR—estimated glomerular filtration rate; GI—gastrointestinal; ICU—intensive care unit; M—month; MPA—mycophenolic acid; RTI—respiratory tract infection; TAC—tacrolimus; UTI—urinary tract infection; WBC—white blood cells.

**Table 6:** The incidence of recurrent infections by etiology and localization.

Recurrent Infections	M1			M1–6			M6–12		
	B	T	*p* Value	B	T	*p* Value	B	T	*p* Value
Recurrent infections in general (%)	5.1	30.8	**0.0033**	66.7	59.0	0.4845	28.2	53.8	**0.0224**
Bacterial infections (%)	0	25.6	**0.0008**	33.3	56.4	**0.0416**	17.9	38.5	**0.0446**
Viral infections (%)	0	2.6	0.3139	10.3	5.1	0.3921	0	7.7	0.0791
Mycotic infections (%)	0	2.6	0.3139	0	2.6	0.3139	0	2.6	0.3139
CMV viremia (%)	0	0	1.0000	10.3	5.1	0.3921	2.6	7.7	0.3113
Multi-drug-resistant bacteria (%)	2.6	20.5	**0.0140**	17.9	41	**0.0261**	17.9	17.9	1.0000
UTI (%)	2.6	23.1	**0.0072**	23.1	46.2	**0.0332**	15.4	33.3	0.0673
RTI (%)	0	2.6	0.3139	2.6	5.1	0.5686	0	0	1.0000
GI infections (%)	0	0	1.0000	5.1	2.6	0.5686	0	5.1	0.1558
Sepsis (%)	0	0	1.0000	0	5.1	0.1558	0	0	1.0000
Other (%)	0	5.1	0.1558	0	10.3	0.3921	0	5.1	0.1558

B—basiliximab; T—thymoglobuline; M—month; CMV—cytomegalovirus; UTI—urinary tract infection; RTI—respiratory tract infection; GI—gastrointestinal.

**Table 7:** Univariate analysis of risk factors for recurrent bacterial infections from first to sixth month after kidney transplantation.

End Point: Recurrent Bacterial Infection M6	OR (95% CI)	*p* Value
Sex (male)	0.6250 (0.2317–1.6857)	0.3518
Age (years)	1.0059 (0.9680–1.0453)	0.7644
Peritoneal dialysis	2.0315 (0.9402–11.6420)	0.9983
Dialysis time (months)	0.9999 (0.9813–1.0189)	0.9941
Duration on WL (days)	0.9992 (0.9978–1.0007)	0.2697
ECD	0.9615 (0.3573–2.5877)	0.9381
PRA actual	0.9469 (0.7558–1.1864)	0.6212
PRA maximal	1.0446 (0.9718–1.1228)	0.1633
CIT (min)	1.0005 (0.9991–1.0019)	0.4843
DGF	6.6129 (0.7348–9.5127)	0.0600
BMI (kg/m^2^)	0.9879 (0.8960–1.0892)	0.8068
Diabetes mellitus	1.7009 (0.9772–2.9606)	0.4010
**Respiratory disease **	5.7200 (1.4508–22.5516)	**0.0062**
Thymoglobulin induction	2.3109 (0.9299–5.7428)	0.0682
Cyclosporine A treatment	1.9480 (0.2307–2.8833)	0.0757
Corticosteroid treatment	0.8944 0.7297–1.0962)	0.2779
ACR	5.2102 (0.7099–38.2415)	0.3037
AHR	3.4037 (0.4360–26.5723)	0.1722
TAC level M1 (ng/mL)	0.8977 (0.7889–1.0216)	0.0891
MPA daily dose M1 (mg/day)	0.9997 (0.9984–1.0009)	0.6200
**Creatinine M1 (µmol/L)**	1.0074 (1.0005–1.0143)	**0.0054**
**eGFR M1 (mL/min)**	0.9739 (0.9479–1.0005)	**0.0448**
WBC M1 (10^9^/L)	0.9857 (0.7915–1.2274)	0.8973
Leukopenia M1	1.6105 (0.6488–3.9977)	0.3029
Platelets M1 (10^9^/L)	1.0019 (0.9933–1.0106)	0.6660
Thrombocytopenia M1	0.6944 (0.2792–1.7276)	0.4323
TAC level M6 (ng/mL)	0.9455 (0.7162–1.2482)	0.6914
MPA dose M6 (mg/day)	0.9981 (0.9969–0.9994)	0.1382
**Creatinine M6 (µmol/L)**	1.0111 (1.0029–1.0193)	**0.0012**
**eGFR M6 (mL/min)**	0.9672 (0.9428–0.9923)	**0.0069**
WBC M6 (10^9^/L)	0.7727 (0.5778–1.0333)	0.0725
Leukopenia M6	1.3986 (0.5443–3.5940)	0.4844
Platelets M6 (10^9^/L)	0.9923 (0.9834–1.0013)	0.0848
**Thrombocytopenia M6**	3.5000 (1.3646–8.9772)	**0.0076**
Need for CSF	1.9451 (0.2288–2.9269)	0.2114
CMV viremia	1.0341 (0.4143–2.5814)	0.9427
**Multi-drug-resistant bacteria**	7.4727 (5.4311–16.2128)	**<0.0001**
**Need for hospitalization**	10.0000 (2.0593–48.5594)	**0.0007**
ICU support	2.0510 (0.8823–3.4215)	0.9981

ACR—acute cellular rejection; AHR—acute humoral rejection; BMI—body mass index; CIT—cold ischemia time; CMV—cytomegalovirus; CSF—colony stimulating factor; DGF—delayed graft function; ECD—extended criteria donor; eGFR—estimated glomerular filtration rate; ICU—intensive care unit; MPA—mycophenolic acid; PRA—panel reactive antibody; TAC—tacrolimus; WBC—white blood cell.

**Table 8:** Multivariate analysis of risk factors for recurrent bacterial infections from first to sixth month after kidney transplantation.

End Point: Recurrent Bacterial Infection M6	OR (95% CI)	*p* Value
**Respiratory tract disease**	3.8640 (1.8384–10.5529)	**0.0108**
Creatinine M1 (µmol/L)	1.0036 (0.9870–1.0204)	0.6750
eGFR M1 (mL/min)	1.0429 (0.9631–1.1293)	0.3012
Creatinine M6 (µmol/L)	1.0020 (0.98771–1.0276)	0.8738
eGFR M6 (mL/min)	0.9762 (0.8930–1.0671)	0.5953
**Thrombocytopenia M6**	9.5904 (1.7012–14.0637)	**0.0104**
**Multi-drug-resistant bacteria**	9.8942 (4.0136–18.6082)	**0.0003**
Need for hospitalization	3.2584 (0.2809–7.7956)	0.3449

M—month; eGFR—estimated glomerular filtration rate.

**Table 9:** Univariate analysis of risk factors for recurrent bacterial infections from sixth to twelfth month after kidney transplantation.

End Point: Recurrent Bacterial Infection M12	OR (95% CI)	*p* Value
Sex (male)	0.7840 (0.2677–2.2961)	0.6590
Age (years)	0.9882 (0.9472–1.0311)	0.5844
BMI (kg/m^2^)	1.0199 (0.9162–1.1353)	0.7193
Thymoglobulin induction	0.3500 (0.1235–0.9919)	0.4569
Cyclosporine A treatment	2.6190 (0.1566–43.8116)	0.5101
Corticosteroid treatment	0.7987 (0.6263–1.0186)	0.0595
ACR	2.0591 (0.5910–7.1743)	0.2274
AHR	0.6190 (0.0653–5.8685)	0.6633
TAC level M6 (ng/mL)	1.0708 (0.7911–1.4494)	0.6584
**MPA dose M6 (mg/day)**	1.9987 (0.9974–2.9999)	**0.0319**
**Creatinine M6 (µmol/L)**	1.0116 (1.0041–1.0194)	**0.0004**
**eGFR M6 (mL/min)**	0.9620 (0.9353–0.9895)	**0.0044**
TAC level M12 (ng/mL)	0.7787 (0.5556–1.0914)	0.0898
**MPA dose M12 (mg/day)**	1.9984 (0.9967–3.0000)	**0.0347**
**Creatinine M12 (µmol/L)**	1.0030 (0.9987–1.0074)	**0.0012**
**eGFR M12 (mL/min)**	0.9550 (0.9303–0.984)	**0.0001**
WBC M6 (10^9^/L)	0.8632(0.6290–1.1821)	0.3483
Leukopenia M6	1.6000 (0.5417–4.7260)	0.3871
Platelets M6 (10^9^/L)	0.9935 (0.9836–1.0035)	0.1919
Thrombocytopenia M6	2.6000 (0.9465–7.1422)	0.0609
WBC M12 (10^9^/L)	0.9404 (0.7192–1.2297)	0.6530
Leukopenia M12	1.0222 (0.3829–3.6810)	0.9732
Platelets M12 (10^9^/L)	0.9990 (0.9890–1.0091)	0.8439
Thrombocytopenia M12	1.9091 (0.6271–5.8122)	0.2609
Need for CSF	2.2667 (0.5477–9.3810)	0.2676
CMV viremia	1.5341 (0.4873–4.8291)	0.4697
**Multi-drug-resistant bacteria**	5.0000 (1.5328–9.6380)	**<0.0001**
**Need for hospitalization**	2.2445 (0.8020–6.1860)	**<0.0001**
ICU support	2.0088 (0.5901–4.2142)	0.2026

ACR—acute cellular rejection; AHR—acute humoral rejection; BMI—body mass index; CMV—cytomegalovirus; CSF—colony stimulating factor; eGFR—estimated glomerular filtration rate; ICU—intensive care unit; MPA—mycophenolic acid; TAC—tacrolimus; WBC—white blood cells.

**Table 10:** Multivariate analysis of risk factors for recurrent bacterial infections from sixth to twelfth month after kidney transplantation.

End Point: Recurrent Bacterial Infection M12	OR (95% CI)	*p* Value
MPA dose M6 (mg/day)	0.9991 (0.9960–1.0021)	0.5641
Creatinine M6 (µmol/L)	1.0102 (0.9914–1.0293)	0.2908
eGFR M6 (mL/min)	1.0528 (0.9462–1.1713)	0.6451
MPA dose (mg/day)	1.0000 (0.9962–1.0037)	0.9869
Creatinine M12 (µmol/L)	1.0000 (0.9924–1.0076)	0.9902
eGFR M12 (mL/min)	0.9754 (0.8874–1.0722)	0.6060
**Multi-drug-resistant bacteria**	8.4263 (1.8690–37.9896)	**0.0055**
Need for hospitalization	4.4173 (3.0509–7.7711)	0.9978

MPA—mycophenolic acid; M—month; eGFR—estimated glomerular filtration rate.

## Data Availability

The data that support the findings of this study are available on request from the corresponding author, G.K.
